# Factor analysis of the Pediatric Symptom Checklist with victimized child laborers in Rural Bangladesh

**DOI:** 10.1192/j.eurpsy.2023.1540

**Published:** 2023-07-19

**Authors:** M. A. Ahad, Y. K. Parry, E. Willis, S. Ullah

**Affiliations:** 1 College of Nursing and Health Sciences; 2 College of Medicine and Public Health, Flinders University, Adelaide, Australia

## Abstract

**Introduction:**

Children in labor are highly vulnerable to intentional maltreatment in the workplace and home environment, particularly in South Asian countries. Since it adversely affects their emotional and cognitive health, it is considered to be a major public health concern. However, the emotional and behavioral consequences of child labor maltreatment are still overlooked.

**Objectives:**

The study investigated the construct validity of a PSC tool for child laborers and the relationship between different maltreatment forms and child laborers’ psychosocial impairments.

**Methods:**

In total, 114 parents of child laborers were recruited using the snowball sampling technique. A structured questionnaire was developed based on three items of the ICAST-P and the parent’s version of the PSC tool. This study performed a factor analysis and a multivariate analysis using SPSS. The data were collected between April and June 2022.

**Results:**

A three-factor model consisting of internalizing, externalizing, and attention problems of child laborers has been partially fitted to the data. The PSC appears to be primarily concerned with internalized psychological difficulties among child laborers, followed by externalized and attention-associated emotional and behavioral difficulties. A mean estimate of the prevalence of maltreatment indicates that child laborers are primarily subjected to psychological maltreatment, followed by physical maltreatment and neglect. The study observed that physically and psychologically maltreated child laborers are significantly screened for psychosocial impairments associated with internalized problems and attention deficits. Psycho-social constructs are not significantly influenced by neglect. There was no significant relationship between maltreatment and externalized psychosocial difficulties among child laborers.

**Conclusions:**

The estimated findings would aid prospective researchers in examining the possible factors associated with the emotional and behavioral problems of maltreated child laborers. In addition, clinicians can gain insight into diagnosing psychometric symptoms in this population of children.

**Disclosure of Interest:**

None Declared

